# Supplementary material for: Trends in the nephrologist workforce in South Africa (2002–2017) and forecasting for 2030
Source: PLoS One. 2021 Aug 12;16(8):e0255903. doi: 10.1371/journal.pone.0255903 (PMC8360377; doi:10.1371/journal.pone.0255903)
Supplement: Attachment — Submitted filename: ResponseToReviewers_20210607.docx [file pone.0255903.s002.docx]

**Responses to reviewer feedback**

Dear Professor Bhimma

Thank you for the constructive critique and for the opportunity to submit a revised manuscript. We list the comments and our responses below.

Thank you and regards
Razeen Davids, on behalf of the authors

**Reviewer and Editor comments:**

1. **Thank you for stating the following in the Acknowledgments Section of your manuscript: "*This study was conducted during the ISN Fellowship of Dominic Kumashie. We gratefully acknowledge the support of the ISN*". We note that you have provided funding information that is not currently declared in your Funding Statement. However, funding information should not appear in the Acknowledgments section or other areas of your manuscript. We will only publish funding information present in the Funding Statement section of the online submission form.

   Please remove any funding-related text from the manuscript and let us know how you would like to update your Funding Statement. Currently, your Funding Statement reads as follows: "The authors received no specific funding for this work." Please include your amended statements within your cover letter; we will change the online submission form on your behalf.**

   Response: Thank you for updating our funding statement to read "*The authors received no specific funding for this work but wish to acknowledge the role of the International Society of Nephrology in providing a nephrology training fellowship for DK.*" We have removed "*International Society of Nephrology Fellow*" from Dr Kumashie’s affiliation details, as requested.
2. **In your ethics statement in the manuscript and in the online submission form, please ensure that you have discussed whether all data/samples were fully anonymized before you accessed them and/or whether the IRB or ethics committee waived the requirement for informed consent.**

   Response: This has been done as requested.
3. **Suggestion: Include (a) statistics from sub-Saharan Africa on the projected increase in the prevalence of chronic kidney disease over the next decade as well as (b) the main causes of chronic kidney disease in the region.**

   Response: We have added a short paragraph (the second paragraph) to the Introduction to address these points. (a) In Africa, the kidney disease burden is likely to increase substantially over the next decade. Contributing factors include high population growth rates, aging of the population and, by 2040, an increase in the number of people with diabetes of 140%. Although most African patients with kidney failure are unable to access KRT because of insufficient resources, the number of people receiving KRT is forecast to increase from 0.083 million in 2010 to 0.236 million by 2030. (b) The most important causes of chronic kidney disease, based on the reported causes of kidney failure in Africans on kidney replacement therapy, are CKD of unknown cause, hypertension and diabetes.
4. **Do the authors feel that nephrologists should also be 'induced' to work in areas currently understaffed, with specific 'inducement allowances' to attract them to those areas?**

   Response: We agree with the principle and have added the following paragraph in the Discussion on this point: Since 2004, a “rural allowance” has been implemented to attract and retain healthcare workers in designated hospitals in underserved, rural areas. For medical specialists like nephrologists, this allowance amounts to 18-22% of their basic salary. Heads of provincial health departments may also include non-rural “inhospitable health institutions” under this agreement. This intervention has been partially effective, with some weaknesses in its implementation and some unintended negative consequences. Nephrology services which include kidney replacement therapy are often based at larger hospitals and these hospitals may not covered by this agreement.

**Additional changes:**

References added for points 3 and 4: refs #5, #6, #20, #21.
